# The Usefulness of Peripheral and Organ Perfusion Monitoring in Predicting Mortality in Patients with Severe SARS-CoV-2

**DOI:** 10.3390/biomedicines13092269

**Published:** 2025-09-15

**Authors:** Mateusz Gutowski, Arkadiusz Lubas, Bartosz Rustecki, Jakub Klimkiewicz

**Affiliations:** 1 Department of Anesthesiology and Intensive Care, Military Institute of Medicine-National Research Institute, 04-141 Warsaw, Poland; 2 Department of Internal Diseases, Nephrology and Dialysis, Military Institute of Medicine-National Research Institute, 04-141 Warsaw, Poland

**Keywords:** perfusion, CRT, mortality, severe COVID-19

## Abstract

**Background**: This study assessed whether repeated monitoring of peripheral and organ perfusion predicts mortality in severe SARS-CoV-2 patients. **Methods**: Peripheral perfusion was measured with finger oxygen saturation (SpO2), capillary refill time (CRT), and finger infrared thermography (FIT). Organ perfusion was measured with the color Doppler renal cortex perfusion (RCP) and Renal Cortical Resistive Index (RCRI). Patients with severe COVID-19 pneumonia were examined after a mean of 7 days of intensive treatment. **Results**: A total of 46 patients (16 women, 30 men, age 55.2 ± 12.7 years) completed the study. SpO2 and CRT emerged as independent key bedside indicators of prognosis, with an OR for death of 0.665 (CI 0.472–0.938) and 2.223 (CI 1.144–4.322). An SpO2 of 95% (sensitivity 58.3%, specificity of 64.7%) and CRT of ≥4 s (sensitivity 66.7%, specificity of 83.9%) were found as the best threshold values for the elevated risk of mortality. From estimated blood tests, only C-reactive proteins (OR 1.252, CI 1.023–1.542) and ferritin (OR 1.001, CI 1.000–1.002) were independently associated with mortality. Moreover, the elevation in CRP was a substantial death indicator (OR 1.707, CI 1.046–2.784). **Conclusions**: The estimation of peripheral perfusion using SpO2 and CRT after initial intensive treatment is helpful in the prediction of outcomes in patients with severe COVID-19.

## 1. Introduction

COVID-19 is a highly contagious disease. It escalated into a global pandemic, causing countless infections and deaths worldwide [1]. This pandemic posed a significant challenge to healthcare systems from low- to high-income countries [1]. Identifying patients at risk for severe disease progression was crucial for prioritizing medical resources and improving survival rates during the pandemic.

COVID-19 is a systemic disease that disrupts physiological function on multiple levels. A generalized immune response may lead to endothelial damage, thrombus formation, and microcirculatory disturbances across every organ [2,3]. An uncontrolled inflammatory response—and, in extreme cases, a cytokine storm—can lead to multi-organ failure, affecting the heart, brain, kidneys, and lungs. Consequently, studies focusing on the monitoring of inflammatory parameters such as cytokines, chemokines, and lymphocyte subpopulations correlated with severe disease progression have emerged [4,5]. Elevated levels of interleukins (ILs) IL-2, IL-7, and IL-10, as well as tumor necrosis factor (TNF), have been shown to be significantly increased in patients with severe COVID-19 cases [6]. For instance, the most common critical complications during deterioration caused by COVID-19 were ARDS, acute cardiac injury, and heart failure [7].

Identifying patients at risk for severe disease progression offers the potential for earlier initiation of intensive treatment and improved prognosis.

Chest computed tomography is considered the gold standard for diagnosing and monitoring COVID-19 and is a valuable method for prognostic assessment [8,9]. Using this tool for patient monitoring may present logistic challenges and pose substantial risks for patients. These risks could arise from transporting unstable patients, transitioning respiratory therapy from stationary to portable systems, alveolar derecruitment, or heightened exposure to X-ray radiation. Therefore, more accessible and effective monitoring methods would be preferable.

Peripheral perfusion can be monitored most commonly with SpO_2_, CRT, and FIT. SpO_2_ is a fundamental vital parameter that is routinely monitored. Oxygen saturation below 90% at hospital admission has been identified as a predictor of mortality in COVID-19 patients [10]. CRT, a component of physical examination, offers valuable insights into microcirculation. Evidence supports its role as an indicator of inadequate organ perfusion and a predictor of mortality [11,12,13]. Sepsis induces sympathetic neurohumoral activation, reducing cutaneous perfusion and lowering skin temperature. Consequently, skin temperature serves as an accessible surrogate for peripheral perfusion and has also been shown to predict mortality [14].

Ultrasound examination proved highly advantageous during the challenging periods of the COVID-19 pandemic. Its portability, ease of transport, straightforward sanitization, safety, and capability for real-time imaging in urgent situations illustrate its value [15]. Point-of-care ultrasound (POCUS) enables real-time monitoring of lung tissue changes, such as the gradual decline in lung capacity and the presence of coalescent B-lines, consolidations, pleural effusion, or pneumothorax. POCUS demonstrates an excellent correlation with CT imaging [16,17]. Likewise, organ perfusion can be examined using various ultrasound techniques, e.g., dynamic tissue perfusion measurement [18,19]. By selecting the region of interest (ROI) in the kidney using a Doppler ultrasound, the volume of blood flow can be assessed [18,19], and a correlation has been observed between the worsening of the oxygenation ratio (PaO_2_/FiO_2_) and perfusion equivalents [20]. Furthermore, inadequate perfusion is associated with deterioration and mortality [20]. This method has been examined in various clinical scenarios [21,22,23,24]. Considering compelling evidence proving that perfusion disturbances are associated with deterioration and mortality, the question arises of whether perfusion monitoring would be useful in assessing patient treatment with COVID-19.

While numerous studies have examined predictors of mortality in COVID-19, there is growing interest in the role of peripheral and organ perfusion parameters. Chatterjee et al. highlighted the prognostic value of low peripheral oxygen saturation (SpO_2_) at admission, demonstrating its association with increased in-hospital mortality among COVID-19 patients, even in cases of “silent hypoxemia” [25]. Similarly, Garcia-Cruz et al. showed that an elevated Renal Resistive Index (RRI), a marker of impaired renal perfusion, was independently associated with acute kidney injury and mortality in critically ill COVID-19 patients [26]. Expanding on this, Gutowski et al. identified capillary refill time (CRT) and renal cortical perfusion (RCP) as independent predictors of deterioration and death, suggesting that a bedside perfusion assessment may offer greater prognostic insight than conventional vital signs alone [27]. These findings underscore the clinical relevance of integrating perfusion monitoring into the management of severe SARS-CoV-2 infections, supporting the rationale for the present study.

This study is distinguished by its innovative approach, which systematically evaluates the prognostic value of peripheral and organ perfusion parameters—specifically SpO_2_, CRT, and renal Doppler indices—in predicting treatment outcomes among patients hospitalized with severe SARS-CoV-2 infections. The novelty of this work lies in its integration of straightforward, bedside-accessible assessments (CRT, SpO_2_) with advanced modalities (renal Doppler and FIT), thereby providing a comprehensive, yet pragmatic, framework for evaluating the perfusion status in critically ill patients.

By prioritizing accessible and non-invasive evaluation methods, this study seeks to improve early risk stratification and facilitate evidence-based treatment decisions, particularly in resource-limited settings. Should these findings be validated in larger cohorts, they hold the potential to inform a broader adoption of perfusion monitoring in COVID-19 care pathways, thereby enhancing prognostic precision and contributing to the reduction in preventable mortality.

## 2. Materials and Methods

### 2.1. Patients

**Figure 1:**
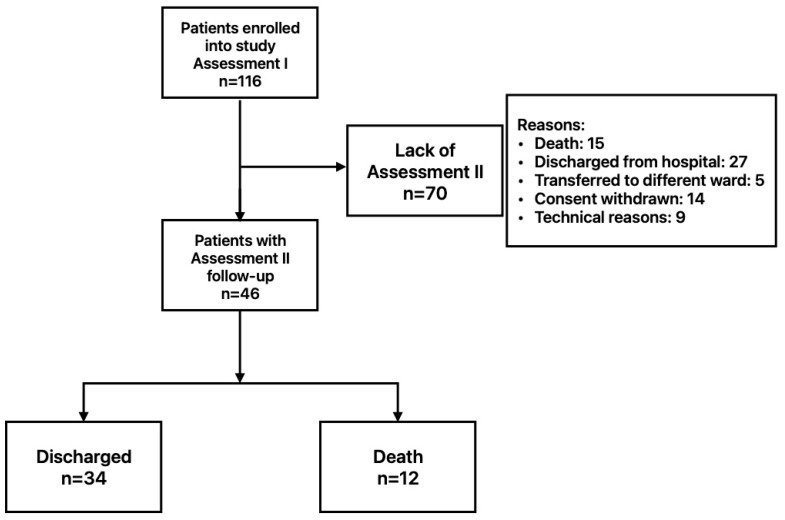
Diagram illustrating the flow of enrolled patients.

A prospective cohort study took place between 2021 and 2022 at the COVID-19 Temporary Hospital of the Military Institute of Medicine in Warsaw, Poland. All participants were patients with severe COVID-19, confirmed with RT-PCR, requiring various forms of oxygen therapy. Severe cases were identified using the following parameters: (1) SpO_2_ levels below 94% on room air; (2) PaO_2_/FiO_2_ ratio under 300 mmHg; (3) respiratory rate over 30 breaths per minute; and (4) lung infiltrates exceeding 50%, measured with CT. Hypoxemia severity was categorized according to the Berlin ARDS criteria into mild (PaO_2_/FiO_2_ between 200 and 300), moderate (100–200), and severe (below 100) [28]. Eligible participants included adults diagnosed with COVID-19, under 70 years old, who could provide informed consent. Participants who were unable to give written informed consent or those who withdrew their consent were excluded from the study. Only patients who had undergone the second peripheral and organ perfusion assessment were included in the study (Figure 1). Adhering to the Helsinki Declaration, the study received approval from the Institutional Bioethics Committee on 19 May 2021 (approval number NR22/WIM/2021).

Building on a projected series of studies evaluating the prognostic utility of perfusion parameters, our previous investigations established that initial assessments of peripheral and organ perfusion served as reliable markers for predicting deterioration and mortality in severe COVID-19 [27]. In the present study, we sought to determine whether repeated assessments of these parameters could further enhance our ability to anticipate the clinical trajectory of the disease. By investigating measurements taken one week after intensive treatment, our objective was to assess the potential additional prognostic value of parameters related to peripheral and organ perfusion.

Patients underwent initial assessments within the first 48 h of their hospital stay. Details regarding their medical history were sourced from existing health records. The evaluations encompassed a physical examination, measurement of capillary refill time (CRT), pulse oximetry to assess oxygen saturation, thermal imaging of the hands using an infrared camera for finger infrared thermography (FIT), and a Doppler ultrasound of the kidneys to analyze dynamic tissue perfusion (DTPM). Data from the DTPM scans were processed after all patient assessments were completed to ensure that the physicians overseeing the patients remained unaware of the results during the study.

The interval between the initial and subsequent peripheral and organ perfusion assessments was approximately seven days, allowing for a comparative analysis of changes in patient status over time.

Prior to hospital admission, all participants received a standard CT scan of their lungs in the emergency department.

All blood analyses were performed in a single laboratory to ensure measurement consistency and data reliability across the study cohort.

### 2.2. Measures

#### 2.2.1. Peripheral Oxygen Saturation—SpO_2_

A peripheral oxygen saturation test was performed using a Sanity Duo Control device (Albert Hohlkörper GmbH & Co. KG, Hemer, Germany) on the fingers of both hands for approximately one minute, with the final result being the average of both measurements.

#### 2.2.2. Capilary Refill Time—CRT

To measure CRT, firm pressure was applied to the ventral surface of the distal phalanx of the second and fourth fingers in both hands until blanching occurred, and this was maintained for five seconds. Using a stopwatch, the examiner recorded the time taken for the skin color to return, averaging all readings.

To ensure consistency across all measurement techniques, a single investigator was responsible for performing each assessment throughout the study, minimizing inter-observer variability and enhancing the reliability of collected data.

#### 2.2.3. Fingertip Infrared Thermography—FIT

For fingertip thermography, an infrared camera FLIR i7^®^ took thermal images from approximately 0.5 m away after the camera had been calibrated for 15 min in the patient’s room. All hand photographs were acquired above the floor to standardize the background and avoid thermal artifacts from surface contact. The temperature of the distal phalanges was then analyzed using Image ThermaBase EU 3.0.0.59 software, providing an average temperature value across all phalanges from both hands.

#### 2.2.4. Oxygenation Ratio—PaO_2_/FiO_2_

The oxygenation ratio (PaO_2_/FiO_2_) is used to determine the severity of respiratory failure in patients with ARDS, as per the Berlin criteria. The classification is based on PaO_2_/FiO_2_ values: scores are 0 for >300, 1 (mild) for 300–200, 2 (moderate) for 200–100, and 3 (severe) for <100 [28].

#### 2.2.5. Ultrasound Examination

A Doppler image of the kidney was acquired with a convex probe, CA2-8AD (2–8 MHz), associated with a Samsung HS40 device (Samsung, Suwon, Republic of Korea). The region of interest (ROI) was defined as the area within the longitudinal cross-section of the kidney cortex located between the outer edges of two pyramids and the kidney capsule, encompassing the interlobular vessels. To ensure image stability, patients were instructed to hold their breath for a few seconds. The color Doppler gain was maintained at a constant, unaltered setting, allowing for consistent image comparison. The flow velocity scale was set to 9.7 cm/s, with minor adjustments made as necessary to optimize flow visualization. Subsequently, a videoclip in the DICOM format covering 3–5 complete cardiac cycles was recorded. The Renal Cortical Resistive Index (RCRI) was calculated with the formula: peak systolic velocity − end-diastolic velocity, divided by peak systolic velocity.

(1) $$Renal\;Cortical\;Resistive\;Index=\frac{Peak\;Systolic\;Velocity-End\text{-}Diastolic\;Velocity}{Peak\;Systolic\;Velocity}$$

Renal cortical perfusion (RCP [cm/s]) represents the average arterial and venous flow intensity (v) in the defined ROI [18].

(2) $$Renal\;Cortical\;Perfusion\left[\frac{\mathrm{cm}}{s}\right]=\frac{v\left[\frac{\mathrm{cm}}{s}\right]*A\left[{\mathrm{cm}}^{2}\right]}{AROI[{\mathrm{cm}}^{2}]}$$

v—arterial and venous flow intensity; A—area; AROI—region of interest area.

This analysis was performed using an external medical software tool (PixelFlux, Chameleon Software, Leipzig, Germany).

### 2.3. Outcomes

The objective of the study was to determine whether peripheral or organ monitoring could predict in-hospital mortality.

### 2.4. Statistical Analysis

The performed test results were presented as means with standard deviations or medians with interquartile range (IQR), depending on meeting the normal distribution criteria. In cases where this criterion was not met, the data were presented as numbers with percentage occurrence. The distribution normality was checked using the Shapiro–Wilk test. Differences between independent nominal variables were tested with the Chi-squared test; otherwise, they were tested with the McNemar test. The *T*-test for independent variables or the Mann–Whitney test was performed to estimate the significance of differences. We used the *T*-test for dependent normally distributed variables or the Wilcoxon’s test to check differences between repeated measurements. Univariable and then multivariable logistic regression analysis was used to evaluate the prognostic properties of the investigated variables. ROC analysis was conducted using the Youden index and an equally high sensitivity and specificity option (EH) [29] to determine the optimal cut-off threshold for mortality prediction. Bootstrapping was used to assess internal validation of variables and confidence intervals. For clinical interpretation, Net Benefit was calculated according to a decision curve analysis [30]. In all analyses, a double-sided *p*-value of <0.05 was considered statistically significant. All statistical tests were calculated using Tibco Statistica v. 13.3 (TIBCO Software Inc., Greenwood Village, CO, USA).

### 2.5. Ethical Considerations

This study was conducted with careful attention to the ethical implications of research involving critically ill patients. All participants provided informed consent prior to enrollment, and the protocol was approved by the institutional bioethics committee. Given that patients with severe COVID-19 constituted a vulnerable population, a thorough risk–benefit analysis was performed at the study’s design stage. The interventions in our study—peripheral perfusion measurements (CRT, SpO_2_ monitoring, thermal imaging) and a Doppler ultrasound of the kidney—were all non-invasive, posed minimal risk, and did not interfere with standard clinical care. These procedures are comparable to routine bedside assessments, ensuring that study participation does not place an undue burden on patients. The potential benefits of the research were significant: by identifying early prognostic indicators of deterioration, this study aimed to inform clinical decision-making and improve triage for severely ill COVID-19 patients, which was a direct benefit for this high-risk group. We adhered to the principles of the Declaration of Helsinki and followed all relevant ethical guidelines to ensure the protection of patient welfare. In situations where patients were temporarily unable to provide consent due to the severity of their illness or the need for intubation, consent was obtained from legally authorized representatives, in line with ethical standards for critical care research. Notably, no experimental treatment was administered as part of this study; all patients received the best available standard of care, and the perfusion assessments were observational. In summary, the study’s design minimized risks and maximized potential benefits, upholding respect for patient autonomy and well-being even in the context of a medical emergency.

## 3. Results

From the initially recruited 116 severe COVID-19 patients, finally, 46 patients’ (16 F, 30 M, age 55.2 ± 12.7) perfusion parameters were evaluated in both assessments [27].

After the first perfusion assessment, 34 patients were discharged, and 12 individuals died. Moreover, in 23 cases, consent to the study was withdrawn, or perfusion estimation was not possible for technical reasons, mainly due to tachypnoea and inability to hold a breath. The baseline characteristics of patients’ perfusion assessments are presented in Table 1.

The median period between Assessment I and Assessment II perfusion assessments was 7 days (IQR 2.0; range 2–14 days). The results of the tests performed in both assessments are presented in Table 2.

From the investigated perfusion parameters, only SpO_2_ and RCRI were substantially different between both assessments.

Statistical analysis revealed significant differences in SpO_2_ and RCRI between the assessments in the study group (93 ± 3 vs. 96 (4), *p* < 0.001 and 0.822 (0.265) vs. 0.941 (0.179), *p* = 0.040, respectively). Conversely, variables such as CRT, FIT, and RCP did not exhibit statistically significant differences.

In the whole investigated group, the following blood tests showed statistically significant differences between assessments: WBC (7.97 (5.56) vs. 9.70 (4.81), *p* = 0.002); hemoglobin (14.0 (3.9) vs. 13.1 (2.3), *p* < 0.001); platelets (197 ± 78 vs. 287 ± 127, *p* < 0.001); CRP (8.1 (7.3) vs. 2.4 (9.8), *p* = 0.004); creatinine (0.9 (0.3) vs. 0.8 (0.3), *p* = 0.004); urea (33.5 (22.0) vs. 43.0 (36.0), *p* < 0.001); AST (51 (38) vs. 33 (40), *p* = 0.022); ALT (35 (23) vs. 53 (52), *p* = 0.006); CK (205.5 (461.0) vs. 176.0 (320.0), *p* = 0.013); and LDH (449 (239) vs. 406 (267), *p* = 0.013).

In deceased patients, significant disparities were observed in hemoglobin levels (13.3 ± 2.7 vs. 12.4 ± 2.1, *p* = 0.047), urea (41.0 (30.5) vs. 66.5 (39.0), *p* = 0.010), and LDH (609.8 ± 230.1 vs. 435.0 ± 134.6, *p* = 0.035).

Among the survivors’ group, the SpO_2_ levels were notably significant (94 ± 3 vs. 96 (3), *p* < 0.001). Additionally, the following blood test result differences were significant: WBC (7.97 (4.90) vs. 9.08 (4.42), *p* = 0.016), hemoglobin (14.4 (3.9) vs. 13.6 (2.4), *p* = 0.004), platelets (203 ± 79 vs. 318 (170), *p* < 0.001), CRP (8.1 (6.3) vs. 1.2 (3.5), *p* < 0.001), creatinine (0.9 (0.3) vs. 0.8 (0.3), *p* < 0.001), urea (28.6 (26.0) vs. 39.0 (26.0), *p* = 0.025), and ALT (36.0 (23.0) vs. 53.5 (48.0), *p* = 0.012).

The differences in the Assessment II results between the deceased and survivors are presented in Table 3. Statistically significant differences between deceased patients and survivors were observed in SpO_2_ levels (92 ± 2 vs. 96 (3) *p* = 0.030) and CRT (4.0 (2.6) vs. 3.0 (1.5) *p* = 0.006). Among the blood test results, significant differences were noted in WBC (10.30 ± 5.50 vs. 9.08 (4.42), *p* = 0.038), CRP (10.2 ± 9.2 vs. 1.2 (3.5), *p* = 0.007), ferritin (1927 (2450) vs. 674 (664), *p* = 0.001), urea (41.0 (30.5) vs. 39.0 (26.0), *p* = 0.010), and the oxygenation ratio (90 (21) vs. 202 (162), *p* < 0.001).

**Figure 2:**
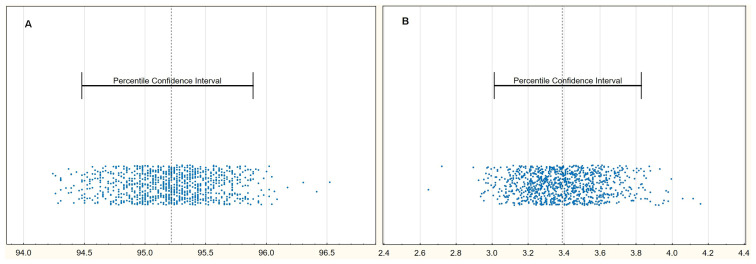
Plots of internal validation via bootstrapping for (**A**): SpO_2_ mean 95.2% (95%CI: 94.5–96.0) and (**B**): CRT mean 3.4 s (95%CI: 3.0–3.8).

Regarding peripheral and organ perfusion parameters, only SpO_2_ and CRT were associated with the risk of death in the univariable analysis (Table 4). Moreover, the same variables appeared to be independently connected with the risk of mortality in the stepwise retrograde multivariable logistic regression analysis, including all investigated perfusion parameters. Plots of internal validation via bootstrapping for SpO_2_ and CRT are presented in Figure 2. There was no missing data in SpO_2_ measurements; however, only 43 of 46 CRT readings were available. Analysis of the missing data shows that it should be classified as missing completely at random (*p* = 0.459).

**Figure 3:**
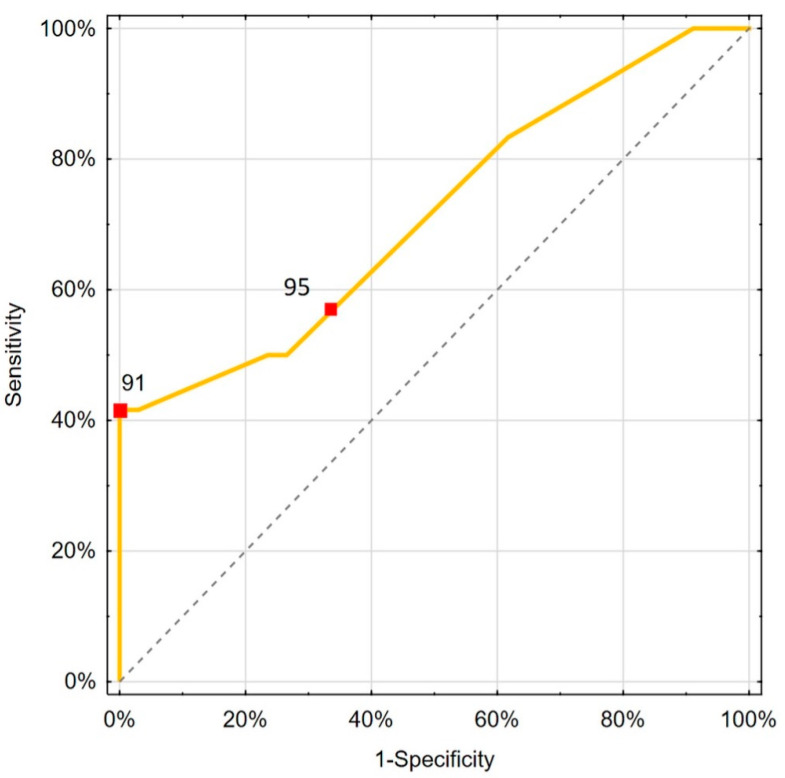
Presentation of saturation ROC analysis. Yellow line: ROC curve of the classifier. Gray dashed line: Reference line for random classification. Red squares with numbers: Selected threshold points on the curve.

**Figure 4:**
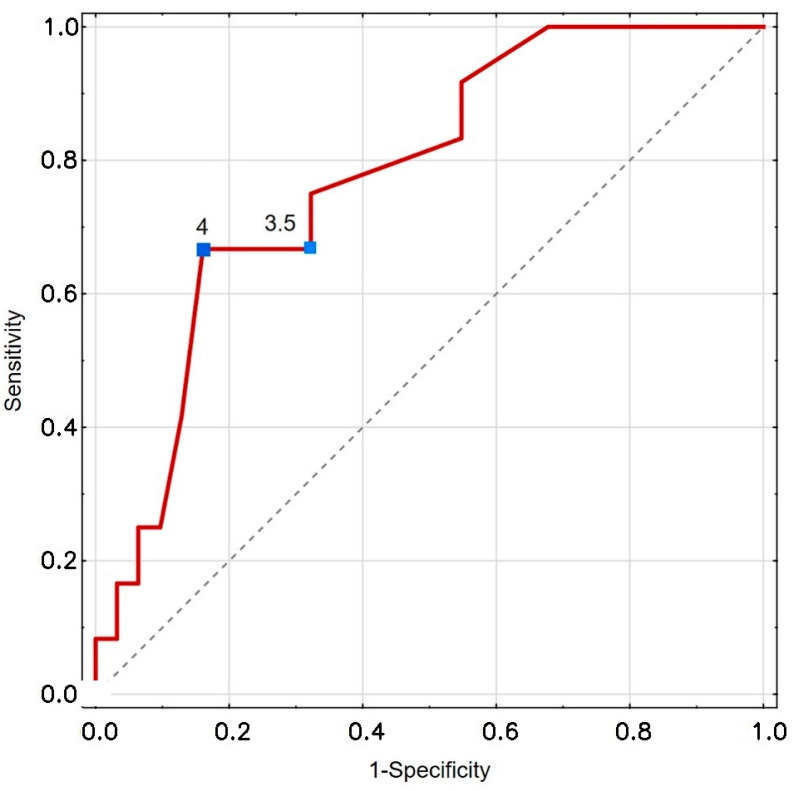
Presentation of CRT ROC analysis. Red line: ROC curve of the classifier. Gray dashed line: Reference line for random classification. Blue squares with numbers: Selected threshold points on the curve.

The values of the proposed cut-off points for PaO_2_ and CRT for mortality risk prediction are shown in Table 5 and Figure 3 and Figure 4.

Considering the calculated thresholds in ROC analysis, results of the decision curve analysis for a threshold probability of 20% are presented in Table 6.

From blood test variables that differed in Assessment II between the deceased and survivors, only CRP and Ferritin were independently associated with the risk of mortality (Table 7). Elevated ferritin remains an independent predictor, though its effect size is small. Higher CRP is independently associated with higher odds of the outcome (per 1 mg/dL increase).

Considering the relative change in results between assessments, only CRP differed significantly between the survivors and deceased (−0.82 (0.56) vs. −0.06 (5.28); *p* = 0.011) and was substantially associated with a bad outcome (OR 1.707; CI 1.046–2.784; *p* = 0.032).

## 4. Discussion

In our study, SpO_2_ showed substantial change over time. Despite oxygen therapy, peripheral oxygen saturation levels were significantly lower in the deceased group than in the survivors in our study, with variations observed between the first and second assessments. Notably, monitoring this parameter is essential, as Mejía et al. identified oxygen saturation below 90% at hospital admission as a risk factor for in-hospital mortality. The oxygenation ratio (PaO_2_/FiO_2_) offers additional insights and has been validated as a valuable monitoring tool. Ratios below 310 were associated with increased mortality [31], reflecting the status of organ function and peripheral perfusion parameters.

Capillary refill time (CRT) was significantly longer in deceased patients (4.4 ± 1.8 s) compared with survivors (3.0 ± 1.0 s). This finding aligns with previous observations that CRT correlates with deterioration and mortality [20,27].

Furthermore, both SpO_2_ and CRT were associated with the risk of death, as confirmed by univariable and multivariable analyses. This is consistent with other studies. In a meta-analysis consisting of four studies involving patients with sepsis and septic shock in the Intensive Care Unit, CRT was found to be a moderate predictor of mortality with a sensitivity of 71.2% and specificity of 73.1% [13]. In sepsis research, prolonged CRT has been shown to correlate with microcirculatory impairment in pediatric populations and to predict mortality strongly [32,33]. Abnormal CRT has also been associated with worse outcomes in patients with hyperlactatemia induced by sepsis [34]. Microcirculatory disturbances in sepsis offer valuable insights into understanding COVID-19-related organ dysfunction. Key features of COVID-19 pathophysiology are a mosaic of respiratory illnesses, microcirculatory disturbances, a hypercoagulability state, a cytokine storm, and a complement system activation [35]. Sepsis-driven microcirculatory disturbances play a critical role in organ dysfunction [36,37]. De Miranda et al. demonstrated that in patients with sepsis and sepsis-acquired acute kidney injury (SA-AKI), the Perfusion Index (PI) and CRT, measured in the first 72 h, were associated with the 28-day mortality rate [12].

In the present study, we presented the discriminatory properties of two predictors (CRT and SpO_2_) of poor prognosis assessed after an average of 7 days of intensive treatment. Given that all patients enrolled in the study received intensive treatment, after the initial treatment period, the question arises of whether such treatment should be continued in all patients, or whether there is a group of patients with a poorer prognosis who could benefit from intensified therapy. In a decision curve analysis, the situation of intensive treatment for all patients reflects a probability of not treating due to the fear of the harmful effects of therapy close to 0% [30]. However, the situation after initial intensive treatment, in which patients received the entire primary course of antiviral therapy and about 70% of the recommended dose of dexamethasone, is less clear. Assuming a 20% chance of not escalating treatment, which often exceeds available recommendations, applying the predictors proposed in our study (Table 6) for therapy intensification may be associated with measurable benefits. For example, for 100 patients with a CRT of ≥4 s, the intervention will additionally benefit an average of 15.7 patients. Patients whose SpO_2_ would be ≤95% will experience slightly less benefits (Net Benefit = 8.7 vs. Net Benefit (treat all) = 7.6). However, the presented analysis concerns situations of uncertainty regarding further treatment and will not apply to maximally intensive treatment of all patients. On the other hand, additional information about unfavorable prognoses may also support the decision-making process regarding the non-escalation of treatment.

In the presented study, finger infrared thermography was not found to be a reliable monitoring tool, as no statistically significant difference was observed between deceased and surviving patients across examinations, and no substantial association with mortality was shown in univariable and multivariable analyses. However, thermography measuring the core-to-skin temperature gradient predicted an 8-day mortality in septic patients [14]. Similarly, Bourcier et al. demonstrated that the toe-to-room temperature gradient reflected tissue perfusion and served as a mortality risk factor [38]. These findings suggest that while skin temperature measurements may provide valuable clinical insights, monitoring finger infrared thermography may not be the optimal method for mortality prediction.

Conversely, nailfold video capillaroscopy (NVC) is increasingly supported by evidence as an effective method for assessing microcirculation in patients with COVID-19 [39,40]. NVC is a non-invasive imaging technique that enables the observation of capillary changes, including alterations in structure, blood flow, and density. While this tool shows significant promise for evaluating diseases affecting microcirculatory vessels, further research is necessary to establish its efficacy.

The Renal Cortical Resistive Index (RCRI) demonstrated a significant increase between the first and second assessments (*p* = 0.040). This trend was observed among deceased patients but did not reach statistical significance (*p* = 0.063), nor was it significant in survivors (*p* = 0.309) or in the comparison between groups during the second assessment (*p* = 0.135).

The RCRI is based on the assessment of arterial perfusion within the renal cortex, while the Renal Resistive Index (RRI) is calculated from interlobar, arcuate, or segmental arteries within the kidney. RRI is widely utilized for the identification of renal damage and is considered a reliable survival indicator in cardiovascular conditions [41].

The Renal Resistive Index (RRI) has been acknowledged for its ability to elucidate the interplay between renal microcirculation and the broader cardiovascular, metabolic, and inflammatory systems [41]. This interaction is attributable to the correlation between the RRI and the Pulsatility (impedance) Index. Consequently, the RRI demonstrates a direct relationship with pulse pressure and an inverse relationship with vascular compliance, or alternatively, a direct relationship with vascular stiffness and impedance [42].

In a clinical context, there is a well-established association between elevated RRI values and acute kidney injury (AKI) [41], including sepsis-associated AKI (SA-AKI) in both adult and pediatric populations [43,44]. Furthermore, high RRI values may play a role in predicting mortality among patients with SA-AKI and in those with severe COVID-19 [26,45].

In patients with COVID-19, an elevated RRI may result from several mechanisms. Glucocorticosteroids can enhance vascular sensitivity to catecholamines, promoting renal arteriolar vasoconstriction, as well as increasing sodium and water retention. Renal hypoperfusion may trigger compensatory vasoconstriction, while COVID-19-associated endotheliitis and microvascular dysfunction, including microthrombi formation in renal capillaries, can further impair blood flow and increase the RRI [46,47].

The Renal Cortical Resistive Index (RCRI) may serve as a prognostic marker, but its predictive value is not established in this group. Larger studies are needed for validation.

Several blood test parameters showed significant variations between the first and second examinations in our study. Notably, CRP and Ferritin were independently related to the risk of death. In general, these markers indicate ongoing inflammation. Leisman et al. found that the immunologic response in COVID-19 closely resembles that seen in septic shock. Accordingly, CRP and Ferritin levels are elevated in COVID-19 patients [48,49]. Currently, numerous studies are investigating the risk factors associated with severe COVID-19. Some studies aim to incorporate Artificial Intelligence (AI) into prediction models. While these models include various parameters, one common factor is the C-reactive protein (CRP) [50]. Shamsoddin’s systematic review of 107 predictive modeling studies summarizes that CRP is a common prognostic marker [51]. Conversely, many studies within this expansive research field do not consider parameters like CRP and Ferritin [52]. On the other hand, persistently elevated inflammatory markers during treatment may indicate their ineffectiveness, potentially explaining the association between such markers and increased mortality.

In addition to its prognostic value, the C-reactive protein (CRP) has recently drawn attention as a potential therapeutic target. Schumann et al. reported early results from the CACOV registry, describing seven critically ill COVID-19 patients treated with CRP apheresis to reduce systemic inflammation [53]. The intervention led to marked reductions in CRP levels and was associated with favorable clinical responses in a subset of patients. Although the cohort was small, the findings suggest that elevated CRP is not only a marker of disease severity but may also play a direct pathogenic role in COVID-19 progression. This supports the clinical relevance of our observation that persistently elevated CRP levels are associated with increased mortality and further emphasizes the importance of integrating inflammatory markers into monitoring protocols.

In the univariable analysis, our results demonstrated that the PaO_2_/FiO_2_ ratio was a significant predictor of mortality (*p* = 0.008). Furthermore, the oxygenation ratio was significantly lower between assessments I and II in the population of deceased patients (*p* = 0.034). These findings align with the established correlation between ARDS severity stages and mortality, as defined by the Berlin criteria [28,54]. It is important to underscore the diverse methodologies employed in predicting mortality based on gas exchange parameters. For instance, Hueda-Zavaleta et al. analyzed the change in PaO_2_/FiO_2_ (ΔPaO_2_/FiO_2_) on initiation of mechanical ventilation and 24 h later in COVID-19 patients, yielding predictive insights regarding patient mortality [55]. Notably, the updated ARDS definition allows the use of SpO_2_/FiO_2_ ratios as equivalents to PaO_2_/FiO_2_, thereby broadening diagnostic applicability in clinical practice [54]. In line with this, Gainstefani et al. employed the ROX index—defined as the ratio of peripheral oxygen saturation (SpO_2_) and a fraction of inspired oxygen (FiO_2_) to respiratory rate—to demonstrate its efficacy in predicting mortality in emergency department settings [56]. The ROX index was also shown to outperform the National Early Warning Score in predicting outcomes for pneumonia patients in emergency departments [57]. Additionally, Chen et al. integrated the oxygenation ratio with positive end-expiratory pressure (PEEP) (PaO_2_/FiO_2_ × PEEP) to demonstrate a nonlinear relationship with in-hospital mortality [58].

In our previous study, we showed that peripheral and organ perfusion were correlated with a lower oxygenation ratio [20].

A notable post-2021 risk model is the ABC2-SPH score (2021), which was developed and validated on thousands of COVID-19 inpatients. This model combines seven readily available predictors—including age, number of comorbidities, C-reactive protein, heart rate, and notably, the SpO_2_/FiO_2_ ratio (a non-invasive oxygenation index)—to predict in-hospital mortality [59]. The inclusion of an oxygen saturation parameter (SpO_2_/FiO_2_) significantly improved prognostic accuracy. In fact, the ABC2-SPH score achieved high discrimination (AUROC ≈ 0.85) and outperformed other scores like ISARIC 4C, underscoring the incremental value of SpO_2_-based monitoring in early mortality risk stratification [59]. This reflects clinical observations that hypoxemia severity (captured by low SpO_2_ relative to O_2_ supply) is strongly linked to COVID-19 outcomes due to the extent of acute respiratory failure in severe cases.

Emerging evidence also highlights microcirculatory dysfunction as a key factor in COVID-19 severity. Capillary refill time (CRT)—a simple bedside index of peripheral perfusion—has been associated with outcomes in critical COVID-19. For example, studies have found that prolonged CRT on ICU admission correlates with higher mortality, even when blood pressure and lactate are normal [60]. In severe COVID-19 ARDS, researchers observed impaired CRT and reduced microvascular flow index [61], indicating significant microvascular compromise despite stable macro-hemodynamics. These findings support a biological mechanism whereby systemic inflammation and endothelial injury from SARS-CoV-2 lead to microvascular thromboses and perfusion shunting, reducing tissue oxygen delivery. Thus, abnormal CRT serves as a clinical marker of this microcirculatory failure, linking peripheral perfusion deficits to the higher risk of organ failure and death in COVID-19. Monitoring CRT alongside SpO_2_ can, therefore, provide complementary insights, with SpO_2_ reflecting pulmonary oxygen exchange and CRT reflecting systemic perfusion—both of which are crucial in predicting mortality in COVID-19 patients.

### Limitations

Although it has promising results, our study has several limitations. First, one important limitation of our study is the relatively small sample size of patients who underwent repeated perfusion measurements. This restriction may considerably reduce the statistical power of our analyses and limit the generalizability of our findings to the broader population of patients with severe COVID-19. As a result, our results should be interpreted with caution, recognizing that the limited cohort size may not adequately capture the heterogeneity seen in clinical practice. To address this concern and ensure that the observed associations can be validated and applied more broadly, future investigations should prioritize the inclusion of larger and more diverse patient cohorts. Such studies will be essential for confirming the reliability and external validity of our findings, thereby enhancing the robustness and applicability of perfusion and inflammatory markers as prognostic tools in severe COVID-19.

Repeated measures were available for only 46 patients, of whom 12 experienced mortalities. The events-per-variable ratio approaches the lower threshold for multivariable modeling, which may increase the risk of overfitting.

RCP/RCRI assessments necessitated breath-holding, leading to the disproportionate exclusion of patients unable to comply. This introduces a risk of systematic missing data and may compromise analyses related to organ perfusion.

Moreover, the spectrum of the inflammatory response is vast, and many more parameters could have been analyzed. For example, cytokines such as IL-6 were not routinely monitored in every patient, resulting in significant data loss. This limitation should be acknowledged as a shortcoming. Future studies should take into consideration cytokine monitoring to provide a more comprehensive understanding of the inflammatory response and its role in COVID-19 outcomes. On the other hand, our study focused on the significance of monitoring perfusion parameters in relation to treatment outcomes. Therefore, we used only routine inflammatory parameters.

The attrition rate in this study—where 23 out of 69 initially eligible patients (approximately 33%) did not complete the second perfusion assessment due to consent withdrawal or technical limitations—introduces a potential source of bias, which could promote more stable patients. These missing data may not be random. As a result, the final analyzed cohort could be skewed toward more stable patients, potentially underestimating the association between perfusion abnormalities and mortalities. This limitation underscores the need for caution when interpreting the results and highlights the importance of future validation in larger, more inclusive cohorts with minimized loss to follow-up.

Another limitation is that the study was designed with only a 7-day follow-up period. While this timeframe provides valuable information on acute changes and early outcomes, it may not fully capture the evolution of microcirculatory abnormalities or the potential for delayed complications. A longer observation window could offer deeper insights into both the short- and long-term consequences of COVID-19 on microcirculation. Extending the follow-up would not only allow for assessment of persistent or resolving perfusion disturbances but would also help elucidate their association with long-term morbidity, organ dysfunction, and recovery trajectories in survivors of severe disease.

## 5. Conclusions

Impaired peripheral and organ perfusion on initial assessment is associated with increased mortality in patients with severe SARS-CoV-2 infection. The principal novelty of this study is the demonstration that repeated assessments of SpO_2_ and capillary refill time (CRT) after one week of intensive treatment reliably predict adverse outcomes in patients with severe COVID-19. From blood tests, the increased concentrations of C-reactive proteins and ferritin in repeated assessments appeared to be promising factors associated with poor outcomes.

Although renal cortical perfusion was initially associated with a risk of deterioration and death, repeated measurements after one week of intensive treatment were not helpful in outcome prediction.

Future studies should aim to externally validate these findings in larger, multicenter, and more diverse patient cohorts to confirm the predictive value and generalizability of SpO_2_ and CRT as prognostic markers. Considering the above-mentioned conclusions, more studies on a larger population are required to better understand the role of perfusion in monitoring patients suffering from severe disease.

## Figures and Tables

**Table 1:** Descriptive data, demography, diseases, and ARDS severity based on oxygenation ratio—PaO_2_/FiO_2_ (n = 46).

Variable		N	%
Gender	Male	30	65.2
	Female	16	34.8
Ward Type	Intensive Care Unit	20	43.5
	High-Dependency Unit	26	56.5
	Death	12	26.1
Comorbidity	Malignancy	5	10.1
	Obesity	7	15.2
	Chronic Kidney Disease	3	6.5
	Coronary Artery Disease	7	15.2
	Heart Failure	2	4.3
	Myocardial infarction	1	2.2
	Atrial fibrillation	6	12.3
	Hypertension	10	21.7
	Diabetes	6	12.3
	Asthma	3	6.5
	Chronic Obstructive Pulmonary Disease	1	2.2
ARDS	No ARDS (PaO_2_/FiO_2_ > 300)	8	17.4
	MILD (PaO_2_/FiO_2_ 300 to 200)	10	21.7
	MODERATE (PaO_2_/FiO_2_ 200 to 100)	19	41.3
	SEVERE (PaO_2_/FiO_2_ < 100)	9	19.6

FiO_2_—fraction of inspired oxygen; PaO_2_—pressure of oxygen; oxygenation ratio—PaO_2_/FiO_2_.

**Table 2:** Comparison of test results between assessments. (**a**) In the study group; (**b**) among the group of deceased patients; and (**c**) among the group of survivor patients.

Variable	Assessment I Mean ± SD Median (IQR)	Assessment II Mean ± SD Median (IQR)	Significance (*p*-Value)
(**a**)			
**Study group (*n* = 46)**			
**Peripheral and organ perfusion parameters**			
SpO_2_ [%]	93 ± 3	96 (4)	<0.001
CRT [s]	3.0 (2.8)	3.0 (1.5)	0.695
FIT [°C]	33.0 (3.9)	32.6 (4.5)	0.702
RCP [cm/s]	0.144 (0.231)	0.108 (0.129)	0.971
RCRI [ratio]	0.822 (0.265)	0.941 (0.179)	0.040
**Blood test results**			
WBC [1 × 10^9^/L]	8.0 (5.6)	9.7 (4.8)	0.002
Hgb [g/dL]	14.0 (3.9)	13.1 (2.3)	<0.001
PLT [1 × 10^9^/L]	197 ± 78	287 ± 127	<0.001
CRP [mg/dL]	8.1 (7.3)	2.4 (9.8)	0.004
Ferritin [ng/dL]	1040.0 (1614.0)	1102.0 (1245.0)	0.405
Creatinine [mg/dL]	0.9 (0.3)	0.8 (0.3)	0.001
Urea [mg/dL]	33.5 (22.0)	43.0 (36.0)	<0.001
AST [U/L]	51 (38)	32 (40)	0.022
ALT [U/L]	35 (23)	53 (52)	0.006
CK [U/L]	206 (461)	176 (320)	0.013
LDH [U/L]	449 (239)	406 (267)	0.013
SBP [mmHg]	127 (13)	121 ± 13	0.154
DBP [mmHg]	77 ± 10	73 ± 11	0.089
PaO_2_/FiO_2_	121 (187)	200 (139)	0.136
(**b**)			
**Deceased group (*n* = 12)**			
**Peripheral and organ perfusion parameters**			
SpO_2_ [%]	92 ± 2	94 (6)	0.230
CRT [s]	4.0 (2.6)	4.0 (1.6)	0.625
FIT [°C]	33.4 (7.8)	33.7 (10.3)	0.919
RCP [cm/s]	0.054 (0.224)	0.081 ± 0.076	0.263
RCRI [ratio]	0.784 (0.308)	1 (0.12)	0.063
**Blood test results**			
WBC [1 × 10^9^/L]	10.3 ± 5.5	13.5 ± 4.6	0.075
Hgb [g/dL]	13.3 ± 2.7	12.4 ± 2.1	0.047
PLT [1 × 10^9^/L]	180 ± 75	219 ± 109	0.155
CRP [mg/dL]	10.2 ± 9.2	12.1 ± 8.7	0.617
Ferritin [ng/dL]	1927 (2450)	2819 ± 1887	1.000
Creatinine [mg/dL]	0.95 (0.40)	0.85 (0.70)	0.433
Urea [mg/dL]	41 (31)	67 (39)	0.010
AST [U/L]	44 (40)	31 (35)	0.248
ALT [U/L]	35 (38)	40 (71)	0.308
CK [U/L]	91 (233)	93 (190)	0.075
LDH [U/L]	609.8 ± 230.1	435.0 ± 134.6	0.035
SBP [mmHg]	131 ± 16	121 ± 17	0.278
DBP [mmHg]	79 ± 10	73 ± 15	0.323
PaO_2_/FiO_2_	90 (21)	98 (50)	0.899
(**c**)			
**Survivor group (*n* = 34)**			
**Peripheral and organ perfusion parameters**			
SpO_2_ [%]	94 ± 3	96 (3)	<0.001
CRT [s]	3.0 (1.8)	3.0 (1.5)	0.422
FIT [°C]	32.8 (3.6)	32.5 (3.8)	0.754
RCP [cm/s]	0.181 (0.266)	0.133 (0.146)	0.387
RCRI [ratio]	0.822 ± 0.129	0.879 (0.230)	0.309
**Blood test results**			
WBC [1 × 10^9^/L]	7.97 (4.90)	9.08 (4.42)	0.016
Hgb [g/dL]	14.4 (3.9)	13.6 (2.4)	0.004
PLT [1 × 10^9^/L]	203 ± 79	318 (170)	<0.001
CRP [mg/dL]	8.1 (6.3)	1.2 (3.5)	<0.001
Ferritin [ng/dL]	806 (925)	674 (664)	0.286
Creatinine [mg/dL]	0.9 (0.3)	0.8 (0.3)	<0.001
Urea [mg/dL]	28.6 (26.0)	39.0 (26.0)	0.025
AST [U/L]	53 (38)	34.5 (54)	0.051
ALT [U/L]	36 (23)	54 (48)	0.012
CK [U/L]	285 (501)	210 (304)	0.071
LDH [U/L]	433 (175)	404 (273)	0.767
SBP [mmHg]	126 (13)	121 ± 12	0.374
DBP [mmHg]	76 ± 9	73 ± 10	0.181
PaO_2_/FiO_2_	192.5 (199)	202 (162)	0.192

WBC—white blood cells; Hgb—hemoglobin; PLT—platelets; CRP—C-reactive protein; AST—aspartate aminotransferase; ALT—alanine transaminase; CK—creatine kinase; LDH—lactate dehydrogenase; SBP—systolic blood pressure; DBP—diastolic blood pressure; CRT—capillary refill time; FIT—finger infrared thermography; RCP—renal cortical perfusion; RCRI—Renal Cortical Resistive Index.

**Table 3:** Comparison of results between deceased and survivors in Assessment II.

Variable	Deceased Mean ± SD Median (IQR)	Survivors Mean ± SD Median (IQR)	Significance (*p*-Value)
**Peripheral and organ perfusion parameters**			
SpO_2_ [%]	92 ± 2	96 (3)	0.030
CRT [s]	4.0 (2.6)	3.0 (1.5)	0.006
FIT [°C]	33.4 (7.8)	32.5 (3.8)	0.550
RCP [cm/s]	0.054 (0.224)	0.133 (0.146)	0.154
RCRI [ratio]	0.784 (0.308)	0.879 (0.23)	0.135
**Blood tests results**			
WBC [1 × 10^9^/L]	10.30 ± 5.50	9.08 (4.42)	0.038
Hgb [g/dL]	13.3 ± 2.7	13.6 (2.4)	0.405
PLT [1 × 10^9^/L]	180 ± 75	318 (170)	0.041
CRP [mg/dL]	10.2 ± 9.2	1.2 (3.5)	0.007
Ferritin [ng/dL]	1927 (2450)	674 (664)	0.001
Creatinine [mg/dL]	0.95 (0.40)	0.80 (0.30)	0.388
Urea [mg/dL]	41.0 (30.5)	39.0 (26.0)	0.010
AST [U/L]	44 (40)	35 (54)	0.881
ALT [U/L]	35 (38)	54 (48)	0.311
CK [U/L]	91 (233)	210 (304)	0.068
LDH [U/L]	609.8 ± 230.1	403.5 (273.0)	0.822
SBP [mmHg]	131 ± 16	121 ± 12	0.993
DBP [mmHg]	79 ± 10	73 ± 10	0.966
PaO_2_/FiO_2_	90 (21)	202 (162)	<0.001

**Table 4:** Results of univariable and multivariable logistic regression for mortality risk analysis, including peripheral and organ perfusion parameters.

Variable	Univariable Analysis			Multivariable Analysis		
	OR	95%CI	*p*	OR	95%CI	*p*
SpO_2_ [%]	0.686	0.512–0.919	0.012	0.665	0.472–0.938	0.020
CRT [s]	2.149	1.136–4.067	0.019	2.223	1.144–4.322	0.018
FIT [°C]	0.991	0.837–1.172	0.912	-	-	-
RCP [cm/s]	0.001	0.000–15.152	0.163	-	-	
RCRI [ratio]	799.7	0.182–3,520,549.7	0.118	-	-	-

**Table 5:** Results of saturation and CRT ROC analysis for mortality prediction.

Variable	Cut-Off Value	Method	Sensitivity (%)	Specificity (%)	LR (−); LR (+)	AUC	Significance (*p*-Value)
SpO_2_ [%]	95	EH	58.3	64.7	0.644; 1.653 0.583; NA	0.714	0.021
	91	Youden	41.7	100.0			
CRT [s]	3.5	EH	66.7	67.7	0.492; 2.067 0.397; 4.133	0.777	<0.001
	4	Youden	66.7	83.9			

AUC—area under curve; EH—cut-off point whose sensitivity and specificity are equally high; LR—likelihood ratio; NA—not available.

**Table 6:** Results of decision curve analysis for threshold probability of 20%.

Variable	Cut-Off Value	Net Benefit (Model)		Net Benefit (Treat All)
		Mean	95%CI	Mean
SpO_2_ [%]	≤95	0.087	−0.022–0.207	0.076
	≤91	0.109	0.022–0.196	
CRT [s]	≥3.5	0.128	0.006–0.262	0.099
	≥4	0.157	0.041–0.285	

**Table 7:** Results of univariable and multivariable logistic regression for mortality risk analysis, including blood test parameters.

Variable	Univariable Analysis			Multivariable Analysis		
	OR	95%CI	*p*	OR	95%CI	*p*
WBC [1 × 10^9^/L]	1.134	0.991–1.297	0.068	-	-	-
PLT [1 × 10^9^/L]	0.993	0.986–1.000	0.046	-	-	-
CRP [mg/dL]	1.081	1.002–1.187	0.044	1.252	1.023–1.532	0.029
Ferritin [ng/dL]	1.001	1.000–1.002	0.014	1.001	1.000–1.002	0.033
Urea [mg/dL]	1.026	1.003–1.049	0.027	-	-	-
PaO_2_/FiO_2_	0.988	0.961–0.993	0.008	-	-	-

## Data Availability

The data presented in this study are openly available in Google Drive at https://drive.google.com/drive/folders/1o1d4ZCXPzDtjjZ-SgeBsycOfvx68YINy?usp=share_link.
